# 20-Hydroxy-3-Oxolupan-28-Oic Acid Attenuates Inflammatory Responses by Regulating PI3K–Akt and MAPKs Signaling Pathways in LPS-Stimulated RAW264.7 Macrophages

**DOI:** 10.3390/molecules24030386

**Published:** 2019-01-22

**Authors:** Yufeng Cao, Fu Li, Yanyan Luo, Liang Zhang, Shuya Lu, Rui Xing, Bingjun Yan, Hongyin Zhang, Weicheng Hu

**Affiliations:** 1 Institute of Life Sciences, Jiangsu University, Zhenjiang 212013, China; 17851567661@163.com; 2 Jiangsu Collaborative Innovation Center of Regional Modern Agriculture & Environmental protection/Jiangsu Key Laboratory for Eco-Agricultural Biotechnology around Hongze Lake, Huaiyin Normal University, Huaian 223300, China; lyy122525@163.com (Y.L.); liangzhang_xj@163.com (L.Z.); lushuyawork@163.com (S.L.); xingrui0707@163.com (R.X.); 15380617203@163.com (B.Y.); 3 Key Laboratory of Mountain Ecological Restoration and Bioresource Utilization and Ecological Restoration Biodiversity Conservation Key Laboratory of Sichuan Province, Chengdu Institute of Biology, Chinese Academy of Sciences, Chengdu 610041, China; lifu@cib.ac.cn; 4 School of Food and Biological Engineering, Jiangsu University, Zhenjiang 212013, China

**Keywords:** inflammation, nitric oxide, macrophage, NF-κB, lupane-type triterpene

## Abstract

20-Hydroxy-3-oxolupan-28-oic acid (HOA), a lupane-type triterpene, was obtained from the leaves of *Mahonia bealei*, which is described in the Chinese Pharmacopeia as a remedy for inflammation and related diseases. The anti-inflammatory mechanisms of HOA, however, have not yet been fully elucidated. Therefore, the objective of this study was to characterize the molecular mechanisms of HOA in lipopolysaccharide (LPS)-stimulated RAW264.7 cells. HOA suppressed the release of nitric oxide (NO), pro-inflammatory cytokine tumor necrosis factor α (TNF-α), and interleukin 6 (IL-6) in LPS-stimulated RAW264.7 macrophages without affecting cell viability. Quantitative real-time reverse-transcription polymerase chain reaction (RT-qPCR) analysis indicated that HOA also suppressed the gene expression of inducible NO synthase (iNOS), TNF-α, and IL-6. Further analyses demonstrated that HOA inhibited the phosphorylation of upstream signaling molecules, including p85, PDK1, Akt, IκBα, ERK, and JNK, as well as the nuclear translocation of nuclear factor κB (NF-κB) p65. Interestingly, HOA had no effect on the LPS-induced nuclear translocation of activator protein 1 (AP-1). Taken together, these results suggest that HOA inhibits the production of cytokine by downregulating iNOS, TNF-α, and IL-6 gene expression via the downregulation of phosphatidylinositol 3-kinase (PI3K)/Akt and mitogen-activated protein kinases (MAPKs), and the inhibition of NF-κB activation. Our findings indicate that HOA could potentially be used as an anti-inflammatory agent for medical use.

## 1. Introduction

The inflammatory response is initiated in living tissues in defense of harmful stimuli, including invading microorganisms, irritants, or noxious chemicals [1]. Physiologically, the inflammatory response provides the benefit of removing invading pathogenic microorganisms, including exogenous stimuli, and promoting the repair of damaged tissues [2,3]. However, prolonged inflammation can lead to long-term, severe inflammatory reactions that can enhance the pathological processes of various inflammatory diseases, such as arthritis, Alzheimer’s disease, septic shock, type 2 diabetes, and cardiovascular diseases [4,5]. Macrophages are major inflammatory and immune effector cells that play crucial roles in producing cytokines, chemokines, and inflammatory mediators, including nitric oxide (NO), prostaglandin E_2_, hydrolytic enzymes, and pro-inflammatory cytokines, such as tumor necrosis factor α (TNF-α), interleukin 1β (IL-1β) and interleukin 6 (IL-6) [6,7]. These pathophysiological changes initiate signal transducers, such as phosphoinositide 3-kinase (PI3K)-Akt, mitogen-activated protein kinases (MAPKs), or Janus kinase/signal transducer and activator of transcription (JAK-STATs) to boost the activation and nuclear translocation of transcription factors, such as nuclear factor κB (NF-κB), activator protein 1 (AP-1), and STATs [8,9].

Any substances that suppress the regulation of these mediators are therefore important for the treatment and prevention of inflammation and related diseases. The pro-inflammatory NF-κB pathway is central to the regulation of inflammation. NF-κB is found to be chronically active in many inflammatory diseases [10,11]. Thus, the development of a potential anti-inflammatory drug derived from natural products based on NF-κB target isolation is a promising avenue for research.

*Mahonia bealei* (Fort.) Carr is a member of the Berberidanceae family and is widely distributed in mountainous areas of southern China. *M. bealei* is included in the Chinese Pharmacopeia as a folk medicine for the treatment of dysentery, jaundice, periodontitis, and bloody urine [12]. Its leaves, which are consumed traditionally in China as a bitter tea, engage in antioxidant, anti-proliferation, anti-inflammatory, anti-bacterial, and anti-influenza activities [13,14,15]. Pharmacological testing of the leaves has been conducted mainly on extracts of the plant and its chemical constituents and their pharmacological activities have yet to be investigated. Previous studies of the active components of this plant have focused primarily on alkaloids, such as epiberberine, berberine, and jatrorrhizine, because they were thought to be responsible for its anti-inflammatory effects [16,17]. However, activity of *M. bealei* non-alkaloids and their underlying mechanisms have yet to be fully defined. 

**Figure 1:**
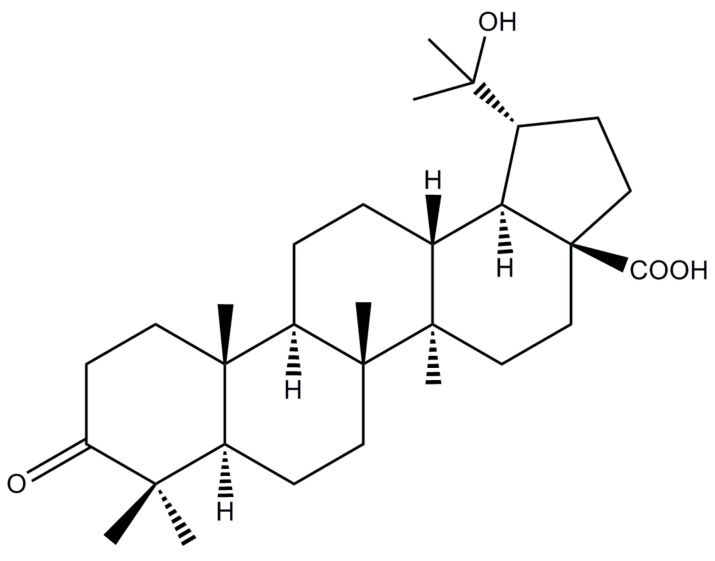
The structure of 20-hydroxy-3-oxolupan-28-oic acid (HOA).

In our previous work, we found that the dichloromethane fraction from *M. bealei* leaves exerted an anti-inflammatory effect both in vitro and in vivo [13]. However, the active compounds in this extract remain unclear. Hence, biological activity guided separation was carried out to search for the active individuals. As a result, a lupane-type triterpene, 20-hydroxy-3-oxolupan-28-oic acid (HOA) (Figure 1) was found to exhibit significant anti-inflammatory effects and NF-κB inhibitory effects (unpublished data). To the best of our knowledge, the biological activities of HOA are unknown. Therefore, as part of our ongoing investigation, this study was conducted to investigate the anti-inflammatory properties and molecular mechanisms underlying the anti-inflammatory properties of HOA.

## 2. Results

### 2.1. Effects of HOA on the Viability of RAW264.7 Cells

**Figure 2:**
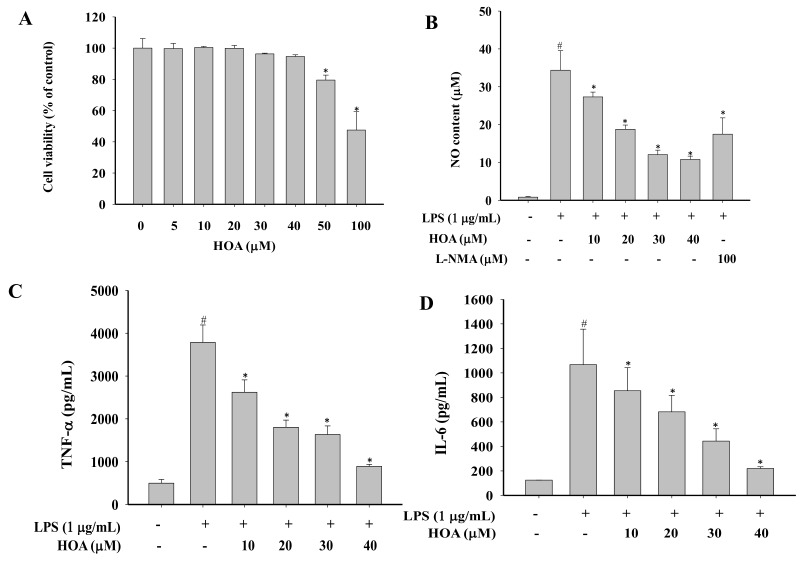
Anti-inflammatory effect of HOA on LPS-induced RAW264.7 cells. (**A**) RAW264.7 cells were treated with various concentrations of HOA for 24 h. The cell viability was determined by MTT assay, as described in section of Materials and Methods. (**B**–**D**) Cells were pretreated with various concentrations of HOA for 30 min and treated with lipopolysaccharide (LPS) for an additional 24 h. The NO content was determined by Griess reagent and the production of cytokines were measured by cytometric bead array (CBA) kit using the flow cytometry. The data are presented as means ± SD (*n* = 3). * indicates a significant difference between LPS group and HOA+LPS groups (*p* < 0.05). ^#^ indicates a difference between LPS group and the control group (*p* < 0.05).

To evaluate the cytotoxic effects of HOA on RAW264.7 cells, cells were incubated with various concentrations of HOA (5, 10, 20, 30, 40, 50, and 100 μM) for 24 h. The result of an MTT assay showed that HOA had no significant cytotoxic effects at concentrations up to 40 μM (Figure 2A). However, cell viability began to decrease to below 90% when the HOA concentration was increased to 50 μM. Accordingly, we limited the concentration of HOA in subsequent experiments to below 50 μM.

### 2.2. Effect of HOA on NO Production and Pro-Inflammatory Cytokine Production in LPS-Stimulated RAW264.7 Cells

In the present study, we first investigated the inhibitory effect of HOA on NO in lipopolysaccharide (LPS)-treated cells. The cells were pre-treated with different concentrations of HOA (10, 20, 30, and 40 μM) for 30 min before adding LPS (1 μg/mL), when a NO detection assay was performed. NO production was 41.76-fold higher in RAW264.7 cells after 24 h of LPS stimulation than in the control group. l-NMMA, an inhibitor of NO that we used as a positive control and also suppressed NO production to 17.46 μM at 100 μM. HOA was found more potent to inhibit NO generation to 27.32, 18.76, 12.06, and 10.79 µM at concentrations of 10, 20, 30, and 40 μM, respectively. In the current study, concentrations of TNF-α and IL-6 in culture supernatants of RAW264.7 cells were detected using a cytometric bead array (CBA) kit. LPS stimulation significantly upregulated the concentrations of pro-inflammatory cytokines (Figure 2C,D). In contrast, treatment with HOA significantly inhibited the levels of TNF-α and IL-6 that were induced by LPS. These results indicate that HOA exerts anti-inflammatory activity via the suppression of NO production and pro-inflammatory cytokines in LPS-stimulated RAW264.7 cells.

### 2.3. Effect of HOA on Morphology of LPS-Stimulated RAW264.7 Cells

**Figure 3:**
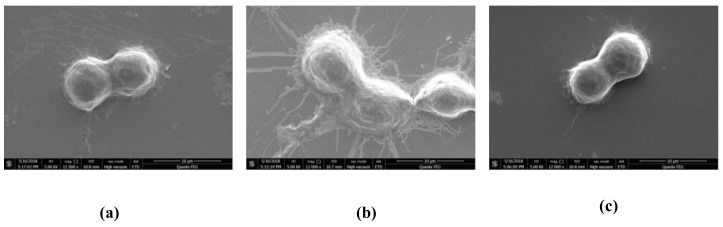
Photograph of RAW264.7 cells after incubation with LPS and HOA under scanning electron microscopy (SEM). (**a**) Control; (**b**) LPS treatment; (**c**) LPS and HOA treatment.

Morphological changes in RAW264.7 cells were assessed with scanning electron microscopy (SEM). The untreated control group RAW264.7 cells were round, with smooth cell edges without pseudopodia (Figure 3), whereas those stimulated with LPS (1 µg/mL) for 10 min had characteristics of activation of macrophage, such as increase in cell size and elongated pseudopodia. Following HOA treatment, the changes in morphological structure of cells were ameliorated. 

### 2.4. Effect of HOA on Expression of Pro-Inflammatory Cytokines in LPS-Stimulated RAW264.7 Cells

**Figure 4:**
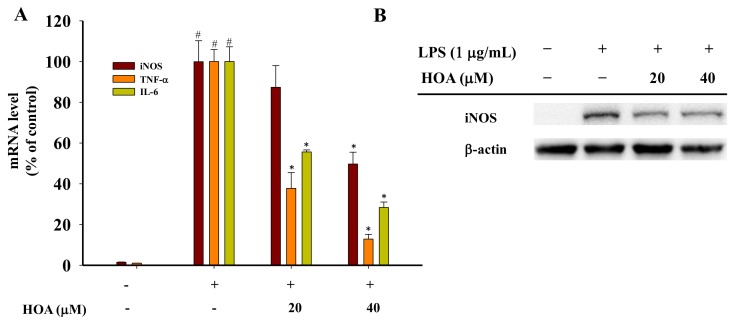
The effect of HOA on LPS-induced pro-inflammatory cytokines expression in RAW264.7 cells. (**A**) Cells were plated at a density of 5 × 10^6^ cells/dish in 60-mm culture dishes and treated with LPS and HOA for 6 h. After preparation of the nuclear fraction, the mRNA expression levels of inducible NO synthase (iNOS), tumor necrosis factor α (TNF-α), and interleukin 6 (IL-6) were measured using reverse-transcription polymerase chain reaction (RT-qPCR). The data are presented as means ± SD (*n* = 3). * indicates a significant difference between LPS group and HOA+LPS groups (*p* < 0.05). ^#^ indicates a significant difference between LPS group and the control group (*p* < 0.05). (**B**) Cells were plated at a density of 5 × 10^6^ cells/dish in 60-mm culture dishes and treated with LPS and HOA for indicated time points. After preparation of the total protein, the expression of iNOS was measured by western blot.

To further determine whether HOA-mediated inhibition of inflammation was involved in the modulation of the inducible NO synthase (iNOS), IL-6, and TNF-α gene expression at the transcriptional level, RAW264.7 cells were pretreated with different concentrations of HOA for 30 min and stimulated with LPS (1 µg/mL) for 6 h and analyzed by reverse-transcription polymerase chain reaction (RT-qPCR). As shown in Figure 4, RT-qPCR revealed that iNOS, IL-6, and TNF-α mRNAs expression levels were low in unstimulated RAW264.7 cells. However, mRNA expression levels were sharply increased by LPS treatment and significantly inhibited by HOA treatment except iNOS expression at 20 μM. Furthermore, western blot was carried out to confirm that iNOS expression was significantly inhibited both at 20 and 40 μM. These results suggest that the inhibitory effect of HOA on iNOS, TNF-α, and IL-6 secretion following LPS stimulation is attributable to downregulation of expression of iNOS, TNF-α, and IL-6.

### 2.5. Effects of HOA on the Regulation of Transcription Factors and Its Upstream Signalling Pathway

**Figure 5:**
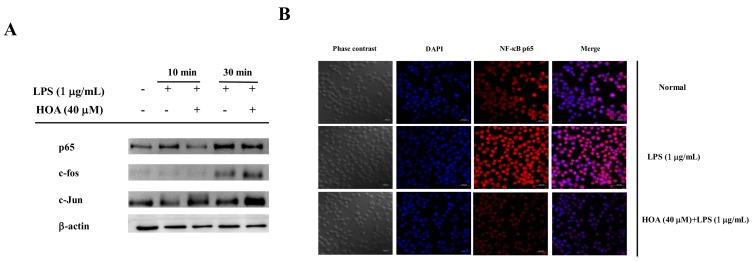
Effect of HOA on translocation of transcription factors in LPS-induced RAW264.7 cells. (**A**) Cells were plated at a density of 5 × 10^6^ cells/dish in 60-mm culture dishes and treated with LPS and HOA for indicated time points. After preparation of the nuclear fraction, the protein expression levels of p65, c-Jun, and c-Fos were measured. (**B**) The localization of NF-κB p65 in the cytoplasm and nuclear were visualized by a confocal microscopy.

**Figure 6:**
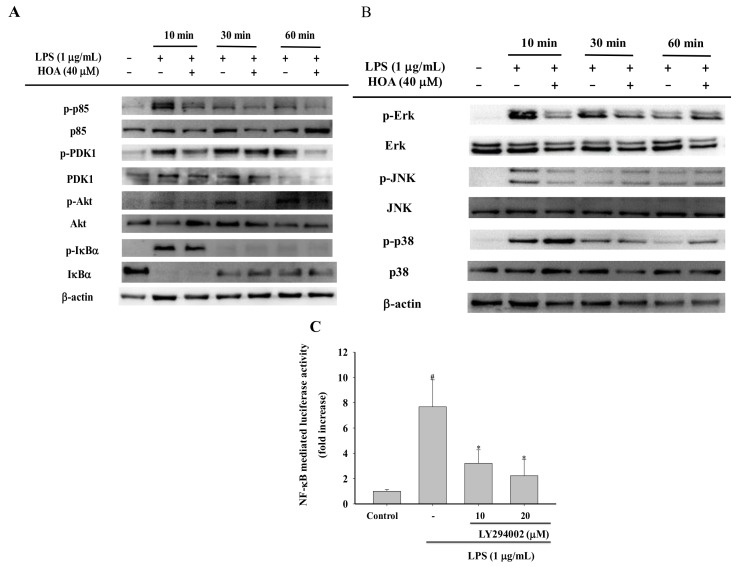
Effects of HOA on LPS-induced activation of PI3K/Akt and MAPKs signaling pathways. (**A**) Cells were plated at a density of 5 × 10^6^ cells/dish in 60-mm culture dishes and treated with LPS and HOA for indicated time points. After preparation of the total protein, the phosphorylated and total forms of PDK1, p85, and Akt were measured by western blot. (**B**) Cells were plated at a density of 5 × 10^6^ cells/dish in 60-mm culture dishes and treated with LPS and HOA for indicated time points. After preparation of the total protein, the phosphorylated and total forms of ERK, JNK, and p38 were measured by western blot. (**C**) Inhibitory effects of LY294002 on LPS-induced NF-κB-luc activity in RAW264.7 cells. Results are representative of three experiments. * indicates a significant difference between LPS group and LY294002 + LPS groups (*p* < 0.05). ^#^ indicates a significant difference between LPS group and the control group (*p* < 0.05).

We examined the effects of HOA on NF-κB/AP-1 translocation into the nuclei of LPS-treated RAW264.7 cells. Macrophages were treated with HOA for 30 min, followed by stimulation with LPS (1 μg/mL) for 10 min and 30 min. HOA suppressed LPS-induced nuclear localization of p65, a major NF-κB subunit, at 10 min and 30 min (Figure 5A). However, HOA did not affect the translation of c-Jun and c-Fos. To further validate the inhibition NF-κB translocation in the LPS-induced inflammatory response, we observed the immunofluorescence intensity of p65 by immunofluorescence staining through a confocal microscope. LPS induced a clear immunofluorescence signal of p65 translocation from the cytoplasm to the nucleus (Figure 5B). Pretreatment with 40 μM HOA dramatically decreased p65 translocation from the cytoplasm to the nucleus. Taken together, these results suggest that HOA attenuates the LPS-stimulated expression of iNOS, TNF-α, and IL-6 by inhibiting NF-κB activation. To assess the modulation of the signaling cascade related to NF-κB expression inhibition, we determined the conserved family of signal transduction enzymes involved in the PI3K/Akt and MAPK pathway. Intriguingly, HOA inhibited the phosphorylation of p85, PDK1, Akt, IκBα, ERK, and JNK in LPS-treated RAW264.7 cells compared with cells treated with LPS alone (Figure 6A,B). We also investigated the functional effect of the PI3K-Akt inhibitor. Pre-incubation of RAW264.7 cells with PI3K-Akt inhibitor significantly inhibited the upregulation of luciferase activity in NF-κB stimulated by LPS in a dose-dependent manner (Figure 6B). 

## 3. Discussion

The pathology of inflammation is initiated by complex processes triggered by microbial pathogens, such as LPS, a prototypical endotoxin that is a component of the outer membranes of gram-negative bacteria [18]. LPS-stimulated macrophages produce reactive nitrogen species (RNS), reactive oxygen species (ROS), and pro-inflammation molecules and cytokines, such as IL-1β, MCP-1, IL-6, and TNF-α, through the TLR4-mediated signaling pathway [19,20]. Therefore, LPS-stimulated TLR4-mediated inflammatory mediators and cytokines have been widely used as excellent models for screening anti-inflammatory drugs and elucidating their underlying mechanisms [21]. The pro-inflammatory NF-κB pathway is central to the regulation of inflammation, and NF-κB is chronically active in many inflammatory diseases [22]. Therefore, it is regarded as a key contributor to the alleviation of inflammatory disorders. To date, numerous natural products have been considered as potential anti-inflammatory agents that can strongly scavenge inflammatory mediators [23,24,25].

Lupane-type triterpenoids are distributed among many plant families. Previous studies have shown that several lupane triterpenes exhibit a wide range of pharmacological effects and engage in important biological activities, especially those involving anti-inflammation, liver protection, anti-tumor processes, and immune system regulation [26,27,28]. Based on our previous activity-guide isolation work, this work was undertaken to clarify the anti-inflammatory potential of HOA on LPS-stimulated RAW264.7 macrophages and its potential mechanisms. 

RNS play an important role in regulating multiple molecular targets related to acute and chronic inflammation [29,30]. NO is endogenous RNS and signaling molecules with a short half-life that is largely released at inflammatory sites and modulates various pathophysiological conditions [31]. Acute and chronic inflammation is induced by NO overproduction, which contributes to the damage of many biological molecules, in turn amplifying inflammation to cause cell death by inducing apoptosis [32]. For these reasons, increased attention is being paid to the development of natural agents for target therapies [33,34,35]. Several pro-inflammatory cytokines, including TNF-α, GM-CSF, IL-6, and IL-1β, are secreted at an early stage and play a critical role in inflammatory-related diseases [36]. TNF-α can regulate the cytokine cascade to stimulate the release of other pro-inflammatory cytokines, such as IL-1β and IL-6, which, in turn, enhance the recruitment of leukocytes to the site of inflammation [37]. IL-6 is a multifunctional cytokine that aggravates the pathogenic processes of autoimmune and inflammatory diseases, such as rheumatoid arthritis, multiple sclerosis, Castleman disease, and fever [38]. Therefore, agents derived from natural compounds that can block the production of these pro-inflammatory mediators could be developed as anti-inflammatory drug candidates. The result of an MTT assay showed that HOA had no significant cytotoxic effects at concentrations up to 40 μM (Figure 2A). Accordingly, the concentrations of HOA used in subsequent experiments were below 50 μM. The present data revealed that HOA significantly inhibited the production of NO, TNF-α, and IL-6 without affecting the cell viability in LPS-stimulated RAW264.7 cells (Figure 2A–D). The secretion of immune-related chemokines and cytokines requires a complicated signaling cascade for the transcriptional activation of inflammatory genes [39]. NO is regulated by three isoforms of NOS, including iNOS, neuronal NOS (nNOS), and endothelial NOS (eNOS). Of these, nNOS and eNOS are related to Ca^2+^/calmodulin activity, whereas iNOS is key enzyme producing an overabundance of NO when induced by bacterial products, such as LPS [40]. Therefore, blocking NO and cytokine production by inhibiting mRNA expression may be a useful approach to the development of novel anti-inflammatory agents [41,42]. In our investigation, we found HOA could significantly inhibit the mRNA expression of iNOS, TNF-α, and IL-6, which was associated with inhibition of the production of NO and proinflammatory cytokines (Figure 4). It was similar to other natural compounds, such as xanthotoxin, cnidilide, and cirsimarin [43,44,45].

NF-κB and AP-1 are ubiquitous transcription factors that are activated during the inflammatory response to LPS to trigger the transcription of pro-inflammatory mediators and associated target genes, such as iNOS, COX-2, and TNF-α [46,47]. Previous literature showed that numerous compounds like poligalen, flavokawain A, and melittin inhibit cytokine production through inhibition nuclear translocation of AP-1 and NF-κB [25,48,49]. Interestingly, we found that HOA could attenuate the nuclear translocation of LPS-induced NF-κB p65 in RAW264.7 cells, whereas it did not affect the nuclear translocation of AP-1 (Figure 5A). The IKK kinase complex is the core element of the NF-κB cascade [50]. As the final confluence point of the inflammation signal, NF-κB participates in the regulation of TNF-α, IL-6, IL-1β, and other inflammatory mediators and plays a very important role in the inflammatory response [30]. In the absence of external stimuli, NF-κB is present in the cytoplasm in an inactive state through the formation of the NF-κB-IκBα complex with IκBs α/β). When cells are stimulated by external conditions, such as LPS, pro-inflammatory factors, and oxidative stress, the conformation of IκBs changes and it is degraded by ATP-dependent proteasomes, releasing Rel protein; a nuclear localization signal then activates NF-κB [51]. Activated NF-κB rapidly translocates into the nucleus after it is detached from IκBα and binds to the target gene κB locus to induce transcription of the target gene [52]. The upstream signaling molecules, including p85/PI3K, PDK1, Akt, ERK, JNK, and p38, are activated by LPS and have been demonstrated to play a vital role in NF-κB activation [53,54]. Additional experiments were carried out to investigate the upstream signaling molecules that could control the activation NF-κB. Similarly, the result showed that inhibiting PI3K can eliminate the phosphorylation of Akt, accompanied by inhibiting the phosphorylation of the proteins from IκBα (Figure 6A) and ultimately inhibiting the activation of NF-κB. Interestingly, the HOA remarkably suppressed the phosphorylation of ERK and JNK at early time point (Figure 6B). These results suggested that HOA inhibits LPS-induced phosphorylation of PI3K/Akt and MAPKs, and activation of NF-κB that similar to other anti-inflammatory agents, such as phlorofucofuroeckol A, cordycepin, and miyabenol A [55,56,57]. 

In summary, our findings showed for the first time that HOA, a lupane-type triterpene, inhibited the LPS-induced release of pro-inflammatory mediators by downregulating the PI3K/Akt and MAPKs signaling pathways. Large quantities of HOA are currently being prepared using a preparative HPLC method, and in vivo efficacy testing is being conducted to further understand the molecular mechanisms of HOA.

## 4. Materials and Methods

### 4.1. Chemicals and Reagents

HOA was isolated from the leaves of *M. bealei* in our lab and the purity of HOA was about 97% determined by high purity liquid chromatography (HPLC) with content of 0.0163% of the dry matter. The spectroscopic data (^1^H-NMR, ^13^C-NMR, and MS data) of HOA were well in accordance with the data reported in the literature [58]. Moreover, (3-4,5-dimethylthiazol-2-yl)-2,5-diphenyltetrazolium bromide (MTT), DAPI, sodium nitrite, dimethyl sulfoxide (DMSO), lipopolysaccharide (LPS), N-1-napthylethylenediamine dihydrochloride, and sulfanilamide were obtained from Sigma-Aldrich (St. Louis, MO, USA). NG-monomethyl-l-arginine (l-NMMA) was available from Beyotime (Haimen, Jiangsu, China). RPMI medium 1640 was purchased from Sigma-Aldrich (Irvine, UK). TRIzol Reagent for isolation of mRNA was obtained from Ambion (Austin, TX, USA). The fetal bovine serum (FBS) was from Corning (Medford, MA, USA). Penicillin–streptomycin solution (10,000 unit/10,000 μg/mL) was purchased from Invitrogen-Gibco (Carlsbad, CA, USA). Prolong Gold anti-fade reagent was obtained from Biomeda (Foster City, CA, USA). Protease and phosphatase inhibitors cocktail tablets were bought from Roche (Mannheim, Germany). SYBR real-time PCR kit was purchased from Bio-Rad (Hercules, CA, USA). The cytometric bead array (CBA) kit was purchased from BD (San Diego, CA, USA). The primary antibodies against p-Erk, Erk, p-JNK, JNK, p-p38, p38, p-PDK1, PDK1, PI3 kinase p85, p-Akt, Akt, p-IκBα, IκBα, c-Jun, c-Fos, NF-κB p65, and COX-2 were purchased from Cell Signalling Technology (Beverly, MA, USA). The other primary antibody including p-PI3 kinase p85/p55 was provided by Abcam (Cambridge, MA, USA). Secondary antibody goat anti-rabbit IgG H&L and goat anti-mouse antibodies were acquired from Abcam (Cambridge, MA, USA). All of the other chemicals and solvents were of analytical reagent grade.

### 4.2. Cell Culture and Cell Cytotoxicity Assay

RAW264.7 cell line was obtained from the American Type Culture Collection (Rockville, MD, USA). RAW264.7 cells were grown in RPMI 1640 medium supplemented with 10% (*v*/*v*) FBS and 1% antibiotics (*v*/*v*) (100 U/mL penicillin 100 μg/mL and streptomycin) and preserved in CO_2_ incubator (SANYO, Tokyo, Japan). Cytotoxicity of HOA against RAW264.7 cells was evaluated by a conventional MTT assay, as previously described. Briefly, cells (1 × 10^6^ cells/well) were cultured for 18 h in a 96-well plate and treated with different concentrations of HOA for an additional 24 h. The optical density (OD) was measured at 550 nm using a microplate reader (Tecan Infinite M200 Pro, Männedorf, Switzerland).

### 4.3. Determination of NO, TNF-α, and IL-6 Content

RAW264.7 macrophage cells were seeded in 96-well plate at a concentration of 1 × 10^6^ cells/mL (100 μL/well) and 18 h. RAW264.7 cells pretreated with HOA or l-NMMA for 30 min were incubated with LPS (1 µg/mL) for 24 h. The NO content was determined by Griess reagent as described previously [59]. The secretion of TNF-α and IL-6 was measured using the flow cytometry (C6 Plus, BD Sceinces, Sparks, MD, USA), according to the manufacturer’s guidelines.

### 4.4. Scanning Electron Microscopy (SEM)

RAW264.7 cells were seeded onto glass coverslips in 6-well plates at a density of 2 × 10^6^ cells/mL (2 mL/well) for 18 h. RAW264.7 cells pretreated with HOA for 30 min were incubated with LPS (1 µg/mL) for 10 min. Hereafter, the cells were fixed in 2.5% glutaraldehyde for 1 h at 4 °C. Next, the cells were washed with PBS three times (5 min each time) and dewatered using 30%, 50%, 70%, 90%, and 100% ethanol gradients two times (5 min each time). The fixed cells were observed with SEM (FEI Quanta 450 FEG, Hillsboro, OR, USA).

### 4.5. Quantitative Real-Time Reverse-Transcription Polymerase Chain Reaction (RT-qPCR) Analysis

RAW264.7 macrophage cells were seeded in 60-mm culture dishes at a density of 5 × 10^6^ cells/well for 18 h. RAW264.7 cells pretreated with HOA for 30 min were incubated with LPS (1 µg/mL) for 6 h. Total RNA was isolated from cells using TRIzol reagent according to the standard protocol. Four micrograms of RNA were reverse transcribed to cDNA using RevertAid First Strand cDNA Synthesis Kit and respective gene expression was determined by CFX-96™ Real-Time instrument (Bio-Rad, Hercules, CA, USA) using the 2^−ΔΔCt^ method. The primers used were from Sangon Biotech (Shanghai, China) and were described in Table 1.

### 4.6. Western Blot Analysis

RAW264.7 macrophage cells were seeded in 60-mm culture dishes at a density of 5 × 10^6^ cells/well for 18 h. RAW264.7 cells pretreated with HOA for 30 min were incubated with LPS (1 µg/mL) for the indicated time points. Commercial kits according to the manufacturer’s instructions prepared the whole cell and nuclear lysates. Western blot analysis was performed as previously reported using the indicated antibodies [60].

### 4.7. Immunofluorescence and Confocal Microscopy

RAW264.7 macrophage cells were seeded sterile cover slips at a concentration of 1 × 10^6^ cells/mL (2 mL/well) and placed in 35 mm petri dishes for 18 h. RAW264.7 cells pretreated with HOA for 30 min were incubated with LPS (1 µg/mL) for 10 min. The cells were washed three times with cold PBS, fixed in 4.0% paraformaldehyde for 15 min, and permeabilized with 0.5% Triton X-100 for 10 min. After that, the cells were blocked with 3% BSA/PBS for 1 h. Cells were then incubated with an NF-κB p65 antibody diluted in 3% BSA/PBS for 2 h. After incubation, cell were washed three times for 5 min with PBS, and incubated with Donkey Anti-Rabbit IgG H&L (Alexa Fluor^®^ 4647) (Abcam, Cambridge, MA, USA) secondary antibody diluted in 3% BSA/PBS for 1 h. Cells were stained with 1 μg/mL DAPI solution and images were captured using a LSM700 confocal laser scanning microscope (Zeiss, Jena, Germany).

### 4.8. Transfections and Luciferase Assay

RAW264.7 macrophage cells were seeded in 12-well plate at a density of 5 × 10^4^ cells/well for 18 h. RAW264.7 cells were then transfected with plasmids containing NF-κB using Lipofectamine 2000 (Invitrogen, Carlsbad, CA, USA). After 24 h, cells were pretreated with LY294002 for 30 min and then incubated with LPS (1 µg/mL) for additional of 24 h. The luciferase activity was carried out using the commercial kit from (Promega, Madison, WI, USA) according to the manufacturer’s instruction.

### 4.9. Data Analysis

All analyses were performed using SPSS 19.0 package (SPSS Inc., Chicago, IL, USA) for Windows 7. Values shown represent the mean ± standard derivation (SD). Differences among samples were compared using a Duncan’s multiple range test and *p*-values less than 5% were considered statistically different.

## Figures and Tables

**Table 1:** The primer sequences for RT-qPCR.

Gene Name	Primer Squence (5′-3′)
*iNOS*	F: CATTGATCTCCGTGACAGCC
	R: CATGCTACTGGAGGTGGGTG
*IL-6*	F: TGGGACTGATGCTGGTGACAAC
	R: AGCCTCCGACTTGTGAAGTGGT
*TNF-α*	F: TGCCTATGTCTCAGCCTCTTC
	R: GAGGCCATTTGGGAACTTCT
*GAPDH*	F: CACTCACGGCAAATTCAACGGCACA
	R: GACTCCACGACATACTCAGCAC

## Abbreviations

AP-1, activator protein 1; CBA, cytometric bead array; DMSO, dimethyl sulfoxide; eNOS, endothelial NOS; FBS, fetal bovine serum; HOA, 20-Hydroxy-3-oxolupan-28-oic acid; HPLC, High Purity Liquid Chromatography; IL-6, interleukin 6; iNOS, inducible NO synthase; IL-1β, interleukin-1β; JAK-STATs, Janus kinase/signal transducer and activator of transcription; l-NMMA, NG-monomethyl-l-arginine; LPS, lipopolysaccharide; MTT, (3-4,5-dimethylthiazol-2-yl)-2,5-diphenyltetrazolium bromide; MAPKs, mitogen-activated protein kinase; NF-κB, nuclear factor-κB; NO, nitric oxide; PGE_2_, prostaglandin E2; PI3K, phosphatidylinositide 3-kinase; RNS, radical nitrogen species; ROS, reactive oxygen species; SEM, scanning electron microscopy; TNF-α, tumor necrosis factor-α.
